# Serum microRNAs as Biomarkers of Human Lymphocyte Activation in Health and Disease

**DOI:** 10.3389/fimmu.2014.00043

**Published:** 2014-02-10

**Authors:** Paola de Candia, Anna Torri, Massimiliano Pagani, Sergio Abrignani

**Affiliations:** ^1^Istituto Nazionale Genetica Molecolare “Romeo ed Enrica Invernizzi,”Milan, Italy

**Keywords:** T lymphocytes, circulating microRNAs, exosomes, vaccination

## Abstract

Induction of the adaptive immune system is evaluated mostly by assessment of serum antibody titers and T lymphocyte responses in peripheral blood, although T and B cell activation occurs in lymphoid tissues. In recent years, the release of microRNAs (miRNAs) in the extra-cellular environment has been exploited to assess cell functions at distance via measurement of serum miRNAs. Activated lymphocytes release a large amount of nano-sized vesicles (exosomes), containing miRNA, however there are insufficient data to determine whether this phenomenon is reflected in modulation of serum miRNAs. Interestingly, miRNA signatures of CD4^+^ T cell-derived exosomes are substantially different from intracellular miRNA signatures of the same cells. We have recently identified serum circulating miR-150 as a sensor of general lymphocyte activation and we strongly believe that miRNAs differentially released by specific CD4^+^ effector T cell subsets (Th1, Th2, Th17, and Treg) may serve as serum biomarkers of their elicitation in lymphoid tissues but also in damaged tissues, potentially providing clinically relevant information about the nature of immune responses in health and disease.

## Blood-Circulating miRNAs as Biomarkers in Health and Disease

MicroRNAs (miRNAs) are small single-stranded RNA molecules (18–25 nt), that once loaded into the Argonaute protein of the silencing complex, pair with messenger RNAs, directly effecting post-transcriptional regulation (1). In recent years, it has been found that most cells release miRNAs in the extra-cellular environment, predominantly in association with either vesicles or protein complexes that protect them from RNAses (2–4). This release can be a passive phenomenon that results from tissue damage, or an active process as in the case of miRNAs actively secreted into the surrounding environment by healthy cells, where once outside, miRNAs can reach the bloodstream and constitute what it is now referred to as the “blood-circulating extra-cellular miRNome.” Extra-cellular miRNAs can be extracted from serum, plasma, and other body fluids and profiled through microarray, real time quantitative PCR or sequencing. This possibility has been exploited to assess cell functions at distance via measurement of serum miRNAs and nowadays blood-circulating miRNAs are regarded among the most promising clinical biomarkers for the diagnosis, prognosis, and therapeutic options of a variety of pathological conditions such as cancer (5–7), cardiovascular diseases (8, 9), diabetes (10), liver pathologies (11, 12), and sepsis (13, 14), among others [reviewed in Ref. (15)].

Circulating miRNAs as clinical biomarkers are not without some technical challenges. First, dilution effects in blood limit the amount of RNA per volume of starting material. Second, cellular detritus, hemolysis, and the presence of contaminating components constitute pre-analytical challenges, potentially impacting reproducibility and sensitivity. Finally, as miRNAs are released by virtually all cells in the body and most of the blood miRNAs are released by large organs as well as highly dividing cells, specificity is impacted by high background. However, the fact that serum miRNAs circulate in different compartments might provide an advantage. miRNAs circulate in association with vesicles of nanometric size (20–100 nm) called exosomes, that are formed by the inward budding and subsequent fusion to the plasma membrane of multivesicular endosomes (16); vesicles of larger size (0.2–1 μm) that bud directly from the plasma membrane, are called microvesicles and comprise also apoptotic and senescent bodies (17); in association with Argonaute protein in a vesicle-free form (3); or linked to high-density lipoproteins (18). This compartmentalization facilitates the purification of specific isolates, enriching for biomarkers of interest. Moreover, the possibility of isolating cell lineage-specific exosomes based upon their parental protein expression patterns, could in principle represent a further advantage in the identification and validation of biomarkers in lymphocyte activation.

## Release of Exosome-Associated miRNAs upon Activation of Lymphocytes: Biological Aspects

The presence of RNA, such as messengers and regulatory RNAs (among which miRNAs) within exosomes was initially described in 2007 (19) but the role of exosomes in conveying intercellular communication had already been extensively investigated in the immune system [reviewed in Ref. (20, 21)]. In 1996, it was first demonstrated that B lymphocytes release exosomes with a significantly different overall surface protein profile from that of the plasma membrane and that they do contain MHC class II and are able to induce antigen-specific MHC class II-restricted T cell responses, demonstrating a specific role for exosomes in antigen presentation *in vivo* (22). Later it was also shown that upon TCR triggering, T lymphocytes produce a large amount of exosomes that bear TCR from the pool of activated complexes. It was suggested that exosomes may work as powerful vehicles to specifically deliver signals to cells with a specific combination of peptide/MHC complexes (23). Furthermore, T lymphocytes have been observed killing target cells by CD95 engagement through membrane CD95L containing exosomes (24). Exosomes can be viewed as communication modules between cells of the immune system, and are regarded by some authors as important to the microenvironment as the release of cytokines and chemokines (25). While the role of intracellular miRNAs has long been recognized as a key level of gene-expression regulation in cells of the immune system (26, 27), its role in exosomes has only begun to be explored. Initial data indicate an exosome-mediated transfer of RNA between T cells and antigen presenting cells during antigen recognition, as well as the capacity of miRNAs transferred during immune synapse to modulate gene-expression in recipient cells (28, 29). To fully elucidate the role of exosome-associated miRNAs, it will be necessary to better characterize the fundamental processes of extra-cellular RNA biogenesis, distribution, uptake, and how they contribute to overall function. In principle, since exosomes contain myriad miRNAs in varying numbers they possess the potential to regulate the expression of multiple genes leading to very effective paracrine control over neighboring cells (30).

## Lymphocyte Signatures of Exosome-Associated miRNAs: A New Landscape to be Unveiled

**Figure 1:**
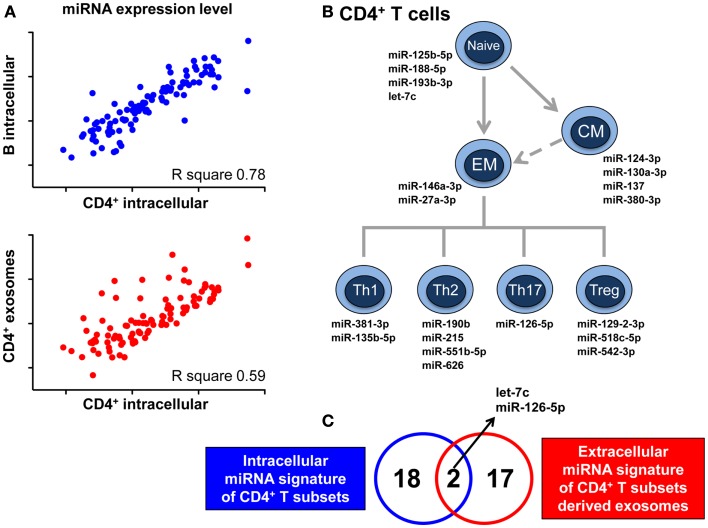
**MicroRNAs signature of CD4^+^ T cell-derived exosomes cannot be predicted by relevant intracellular miRNA signatures of CD4^+^ T cells development and differentiation**. **(A)** Pearson correlation between miRNA expression level in CD4^+^ lymphocytes and either miRNA expression level in B lymphocytes (upper panel) or miRNA representation in CD4^+^ lymphocyte-derived exosomes (lower panel). *R* square values are reported. **(B)** Schematic view of CD4^+^ T lymphocytes with the relative intracellular miRNA signatures identified as relevant for the development and differentiation of these subsets (EM, effector memory; CM, central memory). **(C)** Venn diagram showing the partial overlap between the 20 miRNAs composing the intracellular signatures of CD4^+^ T subsets of panel B and the 19 miRNAs at later stage identified for being either exclusive or differentially up-represented in exosomes derived from CD4^+^ T subsets compared to exosomes derived from B lymphocytes. Data shown are adapted from previous published studies (32, 33).

The release of RNA through exosomes is not a passive phenomenon, but actually a regulated, active process demonstrated by the fact that exosomal RNA content is not at all a mere reflection of that found within the intracellular milieu. By next-generation sequencing of small RNA species present in vesicles released in co-culture of T lymphocytes and dendritic cells, it has been described that distinct RNA categories, such as small ribosomal RNA and specific tRNA fragments, long interspersed elements (LINEs), and long terminal repeats (LTRs) are conveyed in, and released by vesicles in significantly greater numbers than other RNA types, such as lincRNAs (31). A quantitative analysis shows the selective enrichment of some miRNAs in purified exosomes compared to cells. The differential rate of release for various RNA molecules was further demonstrated by our finding that the intracellular miRNome of CD4^+^ T lymphocytes is more similar to the intracellular miRNome of B lymphocytes than to their own exosomal miRNome (Figure 1A) (32). Indeed, we have identified a discrete set of miRNAs whose intracellular concentrations, compared to that found within their cognate lymphocyte-derived exosomes, was significantly different (32).

As previously reported, we have identified 20 intracellular miRNAs that are able to distinguish between different subpopulations of CD4^+^ T cells, defining the development and differentiation of this lineage (Figure 1B) (33). Only two of these miRNAs were independently demonstrated to be differentially represented in Th cell-derived compared to B cell-derived exosomes (Figure 1C); further 17 miRNAs with a signature specifically associated to exosomes released by CD4^+^ T cells have been subsequently identified (32). These observations tell us that even a thorough analysis of the biological relevance of miRNAs at the intracellular level is not helpful in deciphering the spectrum of differences at the extra-cellular level and that only a high-throughput quantitative investigation of miRNA in exosomes will define lymphocyte-specific exosome-associated miRNA signatures. Consequently, this endeavor commenced by showing that CD4^+^ T and B lymphocytes display significantly different selective enrichment of specific extra-cellular miRNAs, and we are now in the process of fully elucidating the differential exosomal miRNomes of each CD4^+^ T subset.

These studies will potentially have two different and equally relevant impacts on the field. On the one side, the analysis of extra-cellular lymphocyte signatures may shed light on the role of miRNA disposal during activation. For example, as previously described, when primary CD4^+^ T cells are activated *in vitro*, they dramatically down-regulate intracellular miR-150 while accumulating it in extra-cellular vesicles, suggesting that this type of release may represent an additional layer of post-transcriptional regulation for miRNAs with very rapid effects on target genes of the discarded miRNAs (32). We thus believe that correlating data of intracellular modulation upon activation with data of extra-cellular disposal will tell us if the case of miR-150 is isolated or not, and if this type of regulation is specific for different lymphocyte subsets. Furthermore, should exosome-associated miRNAs display a paracrine control over neighboring cells, the full description of extra-cellular miRNAs differentially released by different subsets of effector cells will profoundly change the knowledge we have on how these cells impact on the extra-cellular environment.

On the other side, these same studies will pave the way for the identification and validation of potentially powerful biomarkers of lymphocyte activation. Indeed, upon the identification of miRNAs that are differentially released by various lymphocyte effector cells (e.g., Th1, Th2, Th17, and Treg), the assessment of their modulation in serum may render possible to mark the elicitation of these cells, which occurs in lymphoid tissues or damaged organs (34).

## The Growing Need for New Biomarkers of Vaccination

Vaccinations are based on the activation of the adaptive immune system. Their efficacy is evaluated mostly by assessing serum antibody titers and lymphocyte responses in peripheral blood, despite T and B cell activation occurring within the lymphoid tissues. While the protective role of vaccination is primarily conferred by the generation of B cells-derived antigen-specific antibodies, T cells are fundamental for the induction of high-affinity antibodies and immune memory, and in certain types of vaccines, these cells must be regarded as prime effectors. Then, the identification of novel immune correlates of vaccine efficacy that take into account parameters different than antibody titers will become increasingly more important in the development of new adjuvants and the optimization of current vaccines (35). Moreover, as some subsets of effector T helper cells can trigger adverse events from allergy to autoimmunity, the ability to easily monitor the induction of these subsets by new vaccines is becoming critical concern in clinical development.

Toward these ends, a pilot study to address the feasibility of following the generation of an immune response through the profiling of serum-associated miRNAs has been undertaken (32).

## Exosome-Associated miR-150 as a General Sensor of Vaccination

Over the past few years, one specific miRNA, namely miR-150, has been confirmed to play a critical role in the development of lymphoid and myeloid lineages in both mice and humans (36). In particular, miR-150 is expressed at a low level in B- and T-progenitor cells, but gets highly up-regulated in mature lymphocytes. Moreover, as naïve T cells differentiate into effector Th cells, the level of miR-150 is down-modulated again (37).

miR-150 has been also frequently observed to be dramatically dysregulated in several types of leukemias and lymphomas (36). When the level of intracellular miR-150 decreases, some critical targets get de-repressed, e.g., c-Myb, a transcription factor that promotes lymphocyte survival by inducing Bcl2 (38, 39). Another target of miR-150 action is Notch3, a member of the Notch family of receptors, which plays a pivotal role in T cell differentiation and leukemogenesis (40).

We recently described that when primary human CD4^+^ Th cells dramatically down-regulate intracellular miR-150 upon activation, it’s released by exosomes, suggesting that this process of extra-cellular miRNA “disposal” may represent an additional layer of post-transcriptional down-regulation for miRNAs with very rapid effects on target genes that critically control lymphocyte responses (32).

**Figure 2:**
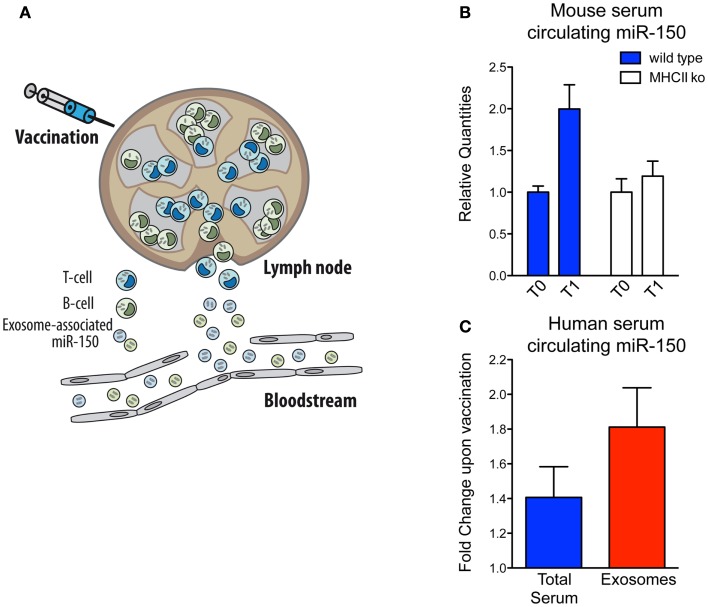
**Serum circulating miR-150 increases in both human and mice upon vaccination**. **(A)** Schematic view of exosome-associated miR-150 release upon induction of adaptive immune response in secondary lymphoid organs upon vaccination. **(B)** Mouse serum circulating miR-150 increase expressed as relative quantities at time T0 (pre-vaccination) and T1 (post-vaccination) in wild type compared to CD4^+^ cell-depleted MHCII knock out mice (four animals/group; the result shown is representative of two independent experiments). **(C)** Human circulating miR-150 increase expressed as fold change upon vaccination in total serum compared to serum purified exosomes (results come from 17 paired samples of sera collected at the time of vaccination and 4 weeks after that). Data shown are adapted from previous published studies (32).

In parallel with the biological implications of these observations, we decided to investigate miRNA-150 as a potential candidate for the optimal biomarker of lymphocyte activation because: (i) it is highly expressed in both human lymphocyte cells and lymphocyte-derived exosomes; (ii) it is expressed specifically in spleen compared to other human tissues, supporting the idea that the major source of serum miR-150 are lymphoid cells and (iii) it is easily detectable and significantly enriched in exosomes circulating in human blood (32). Hence, our working hypothesis is based on the assumption that when the immune system is activated by vaccination, the lymphocytes that participate in the response will release an easily detectable number of exosomes into the bloodstream and consequently a readily measureable level of a lymphocyte-derived exosomal miRNA (as it is the case for miR-150) (Figure 2A). Before proceeding to analyze precious human samples of vaccinated individuals, we made use of the mouse model, and discovered that, as correctly hypothesized, serum miR-150 levels increase significantly in mice upon vaccination with adjuvant-OVA. Instead, miR-150 serum concentrations remain unchanged in immunized mice that are depleted of mature CD4^+^ T lymphocytes [major histocompatibility complex class II-deficient mice (41)] showing that serum miR-150 modulation is a specific phenomenon strictly dependent on adaptive immune responses elicited by effective vaccination (Figure 2B) (32).

Sera from adults or children vaccinated with the 2009 pandemic flu (H1N1) vaccine adjuvanted with MF-59 (42) was then assessed and, similarly to what was found in mice, the level of extra-cellular miR-150 increased significantly in human serum upon vaccination and this increase was significantly more evident upon purification of exosomes (Figure 2C) (32). This observation suggests that serum miR-150 modulation is specifically compartmentalized to lymphocyte-derived vesicles and that exosome purification strategies from blood may serve to increase lymphocyte biomarker sensitivity by enriching immune-related circulating extra-cellular RNAs, as suggested for other conditions (7, 43). Furthermore, in flu vaccinated individuals, miR-150 serum levels post-vaccination have been found to be significantly higher in people mounting higher antibody response, showing a quantitative correlation between the modulation of a circulating miRNA and the adaptive immune response (32).

In a recent study, reduced miR-150 serum concentrations have also been found to be associated with an unfavorable outcome in critically ill patients with sepsis. It has been hypothesized that lower circulating miR-150 levels might lead to de-repression of genes such as CXCR4 and c-Myb, both linked to immune response activation and poor prognosis (14).

Consistently, we do speculate that the significant uptick in circulating miR-150 levels that coincides with vaccination may play a role in down-modulating adaptive immune responses by carrying extra-cellular messages to other immune cells and consequently regulating miR-150 target genes.

Obviously, as miR-150 is ubiquitous across all lymphocyte populations, it may only serve as a generic lymphocyte activation sensor, devoid of more insights into the lymphocyte subsets involved. Nonetheless, this study has provided the first proof-of-concept that serum miRNAs can be readily detected, from a minimally invasive serum sample, toward their validation as sensitive and specific biomarkers of vaccination, and more generally of the adaptive immune response.

## Summary and Perspectives

Blood-circulating extra-cellular miRNAs have the potential to become highly valuable biomarkers in the near future. In particular, the identification of serum miRNA signatures able to directly report the differential activation state of clinically relevant lymphocytic subsets may become an innovative tool to provide pivotal information about the nature of the immune responses occurring in health (e.g., vaccination) and disease (e.g., auto-immune and immune-mediated disorders). We have recently described a significant increase of circulating miR-150 serum concentrations 1 month post-vaccination, and shown to be the release of exosome-associated miRNAs by lymphocytes activated *in vivo* by vaccines. This study gives support to the idea that profiling of serum miRNA levels may lead to the identification of new biomarkers of immune responses and that exosome purification represents a facile yet powerful step toward increased sensitivity, with possible increased specificity.

In conclusion, the compilation of a catalog of exosome-associated miRNAs derived from specific lymphocyte subsets should help confirm whether serum miRNAs are indeed able to report the activation of specific T cell subsets (e.g., Th1, Th2, Th17, Treg, etc.) occurring at distant sites. These lines of enquiry should benefit the assessment of pathogenic immune response during the course of auto-immune diseases and their therapies, as well as significantly contributing to the close, rapid monitoring of clinical trials with new immune-regulatory drugs, new vaccines, and/or adjuvants, particularly in the earlier stages of clinical development.

## Conflict of Interest Statement

The authors declare that the research was conducted in the absence of any commercial or financial relationships that could be construed as a potential conflict of interest.
